# Statistical analysis plan for the ‘Triple Antiplatelets for Reducing Dependency after Ischaemic Stroke’ (TARDIS) trial

**DOI:** 10.1111/ijs.12445

**Published:** 2014-12-30

**Authors:** Philip M W Bath, Katie Robson, Lisa J Woodhouse, Nikola Sprigg, Robert Dineen, Stuart Pocock

**Affiliations:** 1 Stroke Trials Unit, Division of Clinical Neuroscience, University of NottinghamNottingham, UK; 2 Imaging Sciences, Division of Clinical Neuroscience, University of NottinghamNottingham, UK; 3 Department of Medical Statistics, London School of Hygiene and Tropical MedicineLondon, UK

**Keywords:** acute ischemic stroke, acute TIA, aspirin, bleeding, clopidogrel, dipyridamole, recurrence, statistical analysis plan, randomized controlled trial

## Abstract

**Rationale:**

Antiplatelet agents such as aspirin, clopidogrel and dipyridamole are effective in reducing the risk of recurrence after a stroke. Importantly, the risk of recurrence is highest immediately after the index event while antiplatelets cause bleeding.

**Aims and/or hypothesis:**

The ‘Triple Antiplatelets for Reducing Dependency after Ischaemic Stroke’ (TARDIS) trial is testing whether short-term intensive antiplatelet therapy is safe and effective in reducing the early risk of recurrence as compared with standard guideline-based therapy.

**Design:**

TARDIS is an international multi-center prospective randomized open-label blinded–end-point trial, with funding from the UK Health Technology Assessment program. Patients with acute ischemic stroke or transient ischemic attack are randomized within 48 h to intensive/triple antiplatelet therapy or guideline antiplatelets taken for one-month. Patients or relatives give written informed (proxy) consent and all sites have research ethics approval. Analyses will be done by intention-to-treat.

**Study Outcome:**

The primary outcome is shift in stroke recurrent events and their severity, assessed using the modified Rankin Scale, at three-months.

**Discussion:**

This paper and attachment describe the trial's statistical analysis plan, as developed from the protocol during recruitment and prior to unblinding of data. The statistical analysis plan contains design and methods for analyses, and unpopulated tables and figures for the primary and baseline publications. The data from the trial will provide the first large-scale randomized evidence for the use of intensive antiplatelet therapy for preventing recurrence after acute stroke and transient ischemic attack.

Stroke and transient ischemic attack (TIA) are complicated by a high risk of recurrence during the first few hours and days after the index event; a risk that can be mitigated with early and multimodal prophylaxis (1,2). Two mega-trials demonstrated that early antiplatelet therapy with aspirin is effective at reducing recurrence after ischemic stroke (3,4). A meta-analysis of small trials suggested that dual antiplatelet therapy given within 72 h of onset was superior to monotherapy in reducing the early risk of recurrence (5); this systematic review suggested that the composition of antiplatelets (aspirin, clopidogrel, dipyridamole) was less important than using two rather than one agent. Subsequently, the large CHANCE trial found that combined aspirin and clopidogrel was superior to aspirin alone in preventing stroke recurrence by 90 days in Chinese patients with minor ischemic stroke or TIA when randomized within 24 h of onset (6,7).

If dual therapy is superior to monotherapy for acute treatment/prophylaxis, then intensive/triple antiplatelet therapy (aspirin + clopidogrel + dipyridamole) might be better still providing the risk of recurrence is high and bleeding does not become excessive. A series of ‘proof-of-mechanism’ and ‘proof-of-concept’ studies have investigated this approach (8–12). *In vitro* studies found that triple therapy was most effective in inhibiting platelet aggregation, platelet–leucocyte conjugation, and leucocyte activation (8–10). In multiway crossover phase I and II trials comparing short-term administration of mono, dual and triple antiplatelet therapy, the combination of aspirin + clopidogrel, with or without dipyridamole, was most potent in inhibiting platelet function *ex vivo* in both normal volunteers and participants with previous stroke/TIA (11,12). A small parallel group trial of intensive therapy in participants with stroke reported that triple therapy (vs. aspirin alone) was feasible to administer for up to 24 months (13) although there was a nonsignificant trend to increased bleeding with intensive treatment. Chronic triple treatment may be useful in clinical practice in participants at very high risk of recurrence, defined as recurrence on dual antiplatelet therapy (14).

On the basis of these preclinical and clinical data showing feasibility, tolerability and apparent safety of intensive/triple antiplatelets, and the potential for efficacy, the large ‘Triple Antiplatelets for Reducing Dependency after Ischaemic Stroke’ (TARDIS) was started and is ongoing. TARDIS is assessing, in a prospective, randomized, open-label, blinded-outcome design, the safety and efficacy of intensive vs. guideline antiplatelet therapy. The trial commenced in 2009 and will reach 50% of its planned recruitment of 4,100 patients during 2014. The independent Data Monitoring Committee has assessed unblinded data from the trial on eight occasions to date and, on each occasion, recommended that TARDIS should continue.

The accompanying Supporting Information Appendix S1 details the statistical analysis plan (SAP) and is published during recruitment and well before final data cleaning and locking of the trial database so that analyses are not data driven or selectively reported (15). As for the ENOS trial (16), this SAP includes not just information on the planned primary publications but also provides detailed information on the intended baseline characteristics publication.

TARDIS will be reported as both a prevention trial, i.e. efficacy of intensive antiplatelet agents for reducing the frequency and severity of recurrent stroke and TIA (primary aim), and an acute intervention trial, i.e. efficacy in shifting functional outcome. TARDIS is using a novel primary outcome based on both the frequency and severity of recurrent strokes. Conventionally, vascular prevention trials just count recurrent events. However recurrent events may be mild, severe or fatal, and this information can allow ordered categorical outcomes to be defined: fatal event/severe event/moderate event/mild event/no event. Analysis of such polytomous outcomes is more efficient statistically, i.e. they provide improved statistical power for a given sample size, or allow a trial to be smaller for a given power, as shown in an empirical re-analysis of published vascular prevention trials (17,18). This approach follows that used for the design and analysis of trials in acute stroke (19,20). Similarly, adjusted analyses provide additional statistical power (21), are important if minimization is used during the process of randomization (22), and help address any minor imbalances present at baseline due to chance. As a result, these statistical approaches are likely to be more sensitive to any treatment effect and, as such, are recommended by the European Stroke Organisation (23). The use of these approaches, and inclusion of TIA as part of the spectrum of outcomes, allows the size of TARDIS to be almost halved from ∼8000 patients. The collection of all baseline data needed for covariate adjustment of the primary outcome should mean there is no need for imputation for missing data.

Guidelines for use of antiplatelet agents after stroke have changed during the conduct of the trial. At the start of the trial, the UK National Institute for Health and Clinical Excellence (NICE) had recommended the use of aspirin and dipyridamole for secondary prevention (24) and the initial trial protocol defined this as the guideline comparator. However, the widespread availability of inexpensive generic clopidogrel, and significant randomized evidence supporting the use of clopidogrel after stroke, led NICE to update their earlier guidance in 2010 with a recommendation that clopidogrel should be used for secondary prophylaxis. As a result, the protocol was updated and allowed the use of either combined aspirin and dipyridamole, or clopidogrel alone, as the comparator. Investigators are allowed to choose whether randomization includes one or both comparators and this choice can be made separately for stroke and TIA. Choices can be changed but not within 48 h so they cannot influence randomization for a particular patient.

This SAP also informs some of the content of the final trial report to be submitted to the NIHR Health Technology Appraisal (HTA) program; the final report will be submitted for publication in the HTA Journal, part of the NIHR collection of peer-reviewed open access journals. In the future, data from TARDIS will be integrated into individual patient data meta-analyses of antiplatelet agents in acute stroke, and made available to participating countries, and the ‘Virtual International Stroke Trials Archive’ (VISTA) (25). Ultimately, a subset of the data will be made available over the web, as with the International Stroke Trial (26). Similarly, anonymized baseline and on-treatment neuroimaging data will be published (27).

## Supporting Information

Appendix S1 Statistical analysis plan (TARDIS).
