# Supplementary material for: Statistical analysis plan for the ‘Triple Antiplatelets for Reducing Dependency after Ischaemic Stroke’ (TARDIS) trial
Source: Int J Stroke. 2014 Dec 30;10(3):449–51. doi: 10.1111/ijs.12445 (PMC4409839; doi:10.1111/ijs.12445)
Supplement: Appendix S1 — Statistical analysis plan (TARDIS). [file ijs0010-0449-sd1.docx]

STATISTICAL ANALYSIS PLAN

The Triple Antiplatelets for Reducing Dependency after Ischaemic Stroke (TARDIS) trial

Philip M Bath, Katie Robson, Lisa J Woodhouse, Nikola Sprigg, Robert Dineen, Stuart Pocock, on behalf of the TARDIS Trialists

Author for correspondence:

Professor Philip M W Bath, MD FRCPath FRCP

Stroke Trials Unit, Division of Clinical Neuroscience, University of Nottingham, City Hospital campus, Hucknall Road, Nottingham NG5 1PB UK

Tel: +44 115 823 1765

Fax: +44 115 823 1767

E-mail: philip.bath@nottingham.ac.uk


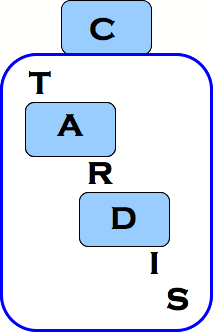


CONTENTS

Appendix A Statistical Analysis Plan

1. Introduction
2. Primary research question
3. Trial design

3.1 Design

3.2 Trial population

3.3 Randomisation

3.3.1 Stratification

3.3.2 Minimisation

3.3.3 Simple randomisation

3.4 Minimising bias

3.5 Ethics and regulatory approvals

4 Statistical analysis plan (SAP)

4.1 Introduction

4.2 Details and caveats

4.2.1 Ordinal events

4.2.2 Ordinal stroke

4.2.3 Modified Rankin Scale

4.2.4 Transient Ischaemic Attack

4.2.5 Other ordinal outcomes

4.3 Sample size

4.4 Outcomes and their analysis

4.4.1 Analysis populations

4.4.1.1 Intention-to-treat safety population

4.4.1.2 Intention-to-treat efficacy population

4.4.1.3 Per protocol population

4.4.1.4 Analyses by population

4.4.2 Missing data, and death

4.4.3 Statistical assumptions

4.4.3.1 Proportionality of odds

4.4.3.2 Alpha spending

4.5 Primary outcome

4.5.1 Measure

4.5.2 Analysis

4.5.3 Covariate adjustment

4.5.4 Subgroup analyses

4.5.5 Sensitivity analyses

4.6 Secondary outcomes

4.6.1 Day 7

4.6.2 Day 35 (end of treatment)

4.6.3 Hospital discharge

4.6.4 Day 90 (end of follow-up)

4.7 Safety analyses – Serious Adverse Events

4.7.1 Death

4.7.2 Bleeding, day 7

4.7.3 Bleeding, day 35

4.7.4 Bleeding, day 90

4.7.5 SAEs, day 7

4.7.6 SAEs, day 35

4.7.7 SAEs, day 90

4.8 Compliance

4.9 Analysis methods

4.9.1 Clinical variables

4.9.2 Imaging variables

4.9.3 Procedures

Appendix B Definitions

1 Definitions of events/outcomes

1.1 Asymptomatic intracerebral haemorrhage

1.2 Bleeding

1.2.1 Major bleeding

1.2.2 Moderate bleeding

1.2.3 Minor bleeding

1.3 Deep vein thrombosis (DVT), symptomatic

1.4 Disposition

1.5 Extracranial haemorrhage, major

1.6 Feeding status

1.7 Headache, requiring treatment

1.8 Intracerebral haemorrhage, secondary on CT/MRI scanning

1.9 Intracranial haemorrhage, symptomatic

1.10 Intracranial haemorrhage

1.11 Myocardial infarction

1.12 Neurological deterioration

1.13 Pulmonary embolism, symptomatic

1.14 Recurrent stroke, symptomatic

1.15 Stroke

1.16 Transient ischaemic attack

1.17 Time at home

1.18 Unstable angina

1.19 Venous thromboembolism, symptomatic

2 Other definitions and notes

2.1 Geographical region

2.2 Haemodynamic variables, calculated

2.3 Acute Stroke Unit

2.4 Stroke Rehabilitation Unit

2.5 Final diagnosis

2.6 Date of death

2.7 EuroQol-5D and Health Utility Status

2.8 Protocol violations

2.9 CT/MR image adjudication

2.9.1 Infarct visibility

2.9.2 Lesion site

2.9.3 Sub-territory sites

2.9.4 Infarcts

2.9.5 Haemorrhages

2.9.6 Other features

Appendix C Primary / NIHR HTA Publication - Tables and figures

Appendix D Secondary publications

Appendix E Baseline publication

REFERENCES

APPENDIX A

A.1 Introduction

The following Statistical Analysis Plan relates to the ‘Triple Antiplatelets for Reducing Dependency after Ischaemic Stroke’ (TARDIS) trial. This study is assessing the safety and efficacy of intensive versus guideline antiplatelet therapy in patients with acute ischaemic stroke or transient ischaemic attack (TIA). The primary outcome is recurrent stroke events and their severity by 90 days post randomisation.

A.2 Primary research question

Is intensive antiplatelet therapy (combined aspirin, clopidogrel and dipyridamole) safe and effective in reducing recurrence and its severity at 3 months, as compared with guideline antiplatelet therapy (clopidogrel, or combined aspirin and dipyridamole), when given acutely after stroke or TIA for one month?

A.3 Trial dESIGN

A.3.1 Design

International collaborative multicentre parallel-group prospective randomised open-label blinded-endpoint controlled phase III trial, with centralised randomisation (stratification and minimisation) to ensure allocation concealment and reduce bias.

A.3.2 Trial population

Previously independent adult patients who are conscious, have an ischaemic stroke or TIA, and are within 48 hours of stroke onset, are eligible for enrolment. The full inclusion-exclusion criteria are given in the protocol.

A.3.3 Randomisation

All patients are randomised to intensive vs guideline antiplatelet therapy. The process of randomisation includes (i) stratification; (ii) minimisation; and then (iii) simple randomisation. Stratification and minimisation allow for improved matching at baseline, stratification allows variable categories to be treated as trials in their own right, minimisation increases statistical power ([1](#_ENREF_1)), and simple randomisation reduces predictability. The stratification and minimisation variables will be used for adjustment of the primary and secondary analyses.

A.3.3.1 Stratification

1. Index event: ischaemic stroke (IS), transient ischaemic attack (TIA)
2. Country
3. Guideline randomisation choice: aspirin/dipyridamole, clopidogrel, either

A.3.3.2 Minimisation (last value is perceived higher risk)

1. Age: <70 years, >70 years
2. Sex: female, male
3. Pre-morbid mRS: 0, >0
4. Time, event to randomisation: >24 hours, <24 hours
5. Number of antiplatelet agents before index event: 0/1, >1
6. Stroke/TIA syndrome: sub-cortical (LACS, POCS), cortical (PACS or TACS) ([2](#_ENREF_2))
7. Systolic BP: <160, >160 mmHg (mean of two measures)
8. Gastroprotection: planned/on, no
9. Use of low dose heparin: no, yes

*In stroke patients*

1. National Institutes of Health Stroke Scale (NIHSS): 0-3, >3
2. Treated with alteplase prior to randomisation: yes, no

*In TIA patients*

1. ABCD2 score: 0-5, >5
2. Number of TIAs in last week: 0/1, >1

A.3.3.3 Simple randomisation

1. On 5% of patients at time of minimisation

A.3.4 Minimising bias

Multiple measures are taken to minimise bias:

1. Central data registration with real-time on-line validation
2. Concealment of allocation
3. Blinded central telephone assessment of 3 month outcome by staff at National Coordinating Centres
4. Assessment of patient recall of treatment ([3](#_ENREF_3))
5. Blinded adjudication of CT scans, SAEs, events and adjudication of events
6. Exclusion of patients enrolled in other trials
7. Analysis by intention-to-treat
8. Adjustment for stratification and minimisation factors, and non-randomised treatment (e.g. alteplase, statins, BP medications)
9. Internet data capture

A.3.5 Ethics and regulatory approvals

TARDIS is run according to the principles of the Declaration of Helsinki and ‘Good Clinical Practice'. The trial was approved by national competent authorities and other bodies (as applicable):

- European Medicines Agency: EudraCT number 2007-006749-42, date 21/11/2007
- UK Medicines & Healthcare products Regulatory Authority (formerly Medicines Control Agency): reference 03057/0027/001-0001, date 17/10/2008
- National Research Ethics Service (NRES, South East Research Ethics Committee): reference 08/H1102/112, date 9/1/2009

The trial is adopted by the:

Australian Stroke Research Network: date 26/9/2012

UK NIHR Stroke Research Network: date 11/3/2009

Trial funding followed external peer review:

- British Heart Foundation: grant PG/08/083/25779, 1 April 2009 – 30 September 2012
- National institutes of Health Research (NIHR) Health Technology Assessment (HTA): grant 10/104/24, 1 October 2012 – 30 September 2017

A.4 Statistical analysis plan (SAP)

¶ Change from protocol version 1.5

A.4.1 Introduction

The trial is designed to recruit 4,100 patients so as to detect a shift in the severity of recurrent stroke, with severity assessed using the modified Rankin Scale (mRS). TARDIS is the first vascular prevention trial to assess recurrence and its severity, rather than recurrence alone. This approach both increases statistical power through comparing the difference in the distribution across the whole scale of severity between the treatment groups ([4](#_ENREF_4), [5](#_ENREF_5)), and allows the effect of treatment on severity to be described. A further, and additional, increase in statistical power is achieved by incorporating key prognostic baseline variables as covariates ([6](#_ENREF_6)).

Hence, the primary analysis of the mRS in those patients who have had a recurrent stroke or TIA will utilise the shift approach, with analysis using ordinal logistic regression including adjustment for covariates.

A.4.2 Details and caveats

A.4.2.1 Ordinal events

TARDIS is the first vascular prevention trial to use ordinal outcomes prospectively for the primary (stroke/TIA), secondary (acute coronary syndromes, composite vascular) and safety (bleeding, adverse events) outcomes. A retrospective analysis of published vascular prophylaxis trials ([4](#_ENREF_4), [5](#_ENREF_5)) and specific assessments of triple antiplatelet therapy ([7](#_ENREF_7)), antihypertensives ([8](#_ENREF_8)), and hormone replacement therapy ([9](#_ENREF_9)), suggest these outcomes give information on both events and their severity, generate smaller numbers-needed-to-treat,([10](#_ENREF_10)) and may be more efficient statistically. Thus, the trial will also test the utility and meaning of ordinal vs binary analyses, thereby adding to its value.

A.4.2.2 Ordinal stroke

The primary outcome comprises a categorical assessment of stroke and TIA ordered by severity, with severity determined by the score on the modified Rankin Scale (mRS):

- Fatal stroke, mRS=6
- Severe non-fatal stroke, mRS 4, 5
- Moderate stroke, mRS 2, 3
- Mild stroke, mRS 0, 1
- TIA
- No stroke or TIA

A.4.2.3 Modified Rankin Scale (mRS)

The modified Rankin Scale (mRS) will be used in participants having a stroke outcome event to determine the severity of that event. The mRS comprises a 7 level ordered categorical scale assessing independence, dependency, and death ([11](#_ENREF_11), [12](#_ENREF_12)):

- - 1. No symptoms at all.
    2. No significant disability, despite symptoms; able to carry out all usual duties and activities.
    3. Slight disability; unable to carry out all previous activities but able to look after own affairs without assistance.
    4. Moderate disability; requiring some help, but able to walk without assistance.
    5. Moderately severe disability; unable to walk without assistance and unable to attend to own bodily needs without assistance.
    6. Severe disability; bedridden, incontinent and requiring constant nursing care and attention.
    7. Dead.

In the context of acute stroke trials, mRS is the standard outcome measure, as recommended by the European Medicines Agency and European Stroke Organisation ([13](#_ENREF_13), [14](#_ENREF_14)). It is sensitive to

- Large treatment effects in small or medium-sized trials: thrombolysis (DIAS, ECASS-3, NINDS, PROACT ([15-18](#_ENREF_15))), factor VIIa ([19](#_ENREF_19))
- Modest treatment effects in large trials: thrombolysis (IST-3 ([20](#_ENREF_20))), blood pressure lowering (SCAST, INTERACT-2 ([21](#_ENREF_21), [22](#_ENREF_22)))
- Small treatment effects when used in mega-trials: aspirin (IST, which used the ‘3 questions’, 3Q, version of the mRS ([23](#_ENREF_23)))

As with analysis of the mRS in acute stroke trials, the comparison will assess the shift in severity of outcome between the treatment groups. The choice of cuts in mRS divides participants into those with a mild event who have made an excellent outcome (mRS 0, 1), those with a moderate event (mRS 2, 3), those with a severe event (mRS 4, 5) and those who died (mRS 6).

Although some previous studies (e.g. SAINT-I/II, ECASS-3 ([18](#_ENREF_18), [24](#_ENREF_24), [25](#_ENREF_25))) combined death with severe disability, i.e. combining mRS categories 5 and 6, most trials have separated these since some treatments, at least theoretically, might influence mortality and disability separately. TARDIS will keep mRS categories 5 and 6 separate.

A.4.2.4 Transient Ischaemic Attack (TIA)

It is important that the full range of severity of recurrence is examined, with TIA forming the mild end of the spectrum. TIA has been a component of the primary outcome embedded within recurrent stroke or vascular events in previous trials (e.g. EARLY ([26](#_ENREF_26))) and meta-analysis (e.g. FASTER ([27](#_ENREF_27))). Reported TIAs are adjudicated independently of the recruiting investigator and blinded to treatment. In a secondary analysis, we will also assess the effect of treatment on stroke without including TIA in the spectrum of severity.

A.4.2.5 Other ordinal outcomes

Other events will also be analysed as ordinal outcomes:

- Acute coronary syndrome: Fatal / MI + heart failure / MI / unstable angina (UA) / none
- Composite vascular: Fatal / non-fatal stroke or MI / TIA or UA / none
- Symptomatic venous thromboembolism: Fatal / PE / DVT / None
- Disposition at hospital discharge: Death / institution / home
- Bleeding: Fatal / severe non-fatal / moderate / mild / no bleeding
- Adverse events: Fatal / serious non-fatal / selected adverse event / no adverse event

A.4.3 Sample size ¶

TARDIS was designed with a start-up phase to assess safety, feasibility and tolerability (funded by British Heart Foundation) and a main phase to assess safety and efficacy (funded by Health Technology Assessment).

The null hypothesis (H_0_) is that intensive antiplatelets will not alter the frequency and severity of stroke/TIA in participants with previous ischaemic stroke or TIA. The alternative hypothesis is that the frequency and severity of stroke/TIA differ between those participants randomised to intensive versus guideline antiplatelets. A total sample size ([28](#_ENREF_28), [29](#_ENREF_29)) of 4,100 (2,050 per group) participants with ischaemic stroke or TIA is required, assuming overall significance (alpha) = 0.05; power (1-beta) = 0.90; odds ratio of 0.68 (equivalent to an odds ratio of 0.57 and relative risk reduction = 0.31 for binary stroke); distribution in outcome as below; treatment crossovers 5.0% and losses to follow-up 2%; and a reduction of 20% for baseline covariate adjustment.([6](#_ENREF_6))

Originally a 5-level stroke/TIA ordered outcome was planned with distribution based on early blinded-TARDIS data (N=392):

- Fatal stroke, 0.51% / mRS 2-5, 0.77% / mRS 0 or 1, 1.53% / TIA, 3.57% / no event, 93.62%

To allow discrimination of moderate and severe non-fatal stroke outcomes, and to keep together mRS scores of 2 and 3 (which can be challenging to separate clinically), a 6-level scale will now be used. Blinded data from the last Trial Steering Committee report suggest the following overall outcome distribution (based on N=1,460 participants):

- Fatal stroke, 0.55% / mRS 4 or 5, 0.55% / mRS 2 or 3, 1.30% / mRS 0 or 1, 1.23% / TIA, 3.22% / no event, 93.15%

A.4.4 Outcomes and their analysis

All outcomes will be assessed with comparison of intensive antiplatelets versus guideline antiplatelets.

A.4.4.1 Analysis populations

The following populations of patients are defined for analyses and take account that the trial is open-label in design.

*A.4.4.1.1 Intention-to-treat safety population*

All randomised participants with vital status recorded. This population will be reported in the primary publication.

*A.4.4.1.2 Intention-to-treat efficacy population*

All randomised participants who received at least one antiplatelet treatment post-randomisation and who have the primary outcome (recurrence and its severity assessed using the mRS) recorded. This population will be reported in the primary publication.

*A.4.4.1.3 Per protocol population*

All randomised participants who received at least 7 days of randomised antiplatelet treatment (or who died during this period), and who have the primary outcome recorded, and who did not have a relevant protocol violation as defined in *Appendix B.2.8*.

*A.4.4.1.4 Analyses by population*

All efficacy analyses will be performed on the intention to treat population. A separate ‘per protocol’ analysis will be performed on the primary and key secondary outcomes. Safety analyses will be performed on the safety population.

A.4.4.2 Missing data, and death

Missing data will not be imputed. Participants who die will be assigned discrete values for outcome measures with a value worse than any living value (as is standard for mRS and BI) ([30](#_ENREF_30)). This ensures that patients who die are included in all analyses, and avoids giving death the same value as the worst possible living outcome score. The scores for the various outcome scales are given with ‘best to worst’ and then the value for death:

- Modified Rankin Scale (mRS): 0 to 5 with death = 6
- Barthel Index (BI): 100 to 0 with death = -5
- National Institutes of Health Stroke Scale (NIHSS): 0 to 42 with death = 43
- EuroQoL-5 dimensions (EQ-5D) as health utility status (HUS): 1 to -0.594 with death = 0 ([30](#_ENREF_30), [31](#_ENREF_31))
- EuroQoL Visual Analogue Scale (EQ-VAS): 100 to 0 with death = -1
- Telephone Interview for Cognitive Status (TICS): 37 to 0 with death = -1
- Telephone Mini Mental Status Exam (t-MMSE): 18 to 0, with death = -1
- Verbal fluency (animal naming): ∞ to 0, with death = -1
- Zung Depression Scale (ZDS): 25 to 100 with death = 102.5

Patients who are still in hospital at day 90 will have a length of stay assigned at 110 days. Similarly, death will be censored at day 110.

A.4.4.3 Statistical assumptions

*A.4.4.3.1 Proportionality of odds*

The treatment effects on ordered categorical data will be compared using ordinal logistic regression (OLR) with adjustment for the prognostic covariates as listed in 3.3, plus use of alteplase. OLR assumes proportionality of odds and this will be tested using a likelihood ratio test. If the proportional odds assumption is not met, OLR will still be used and the lack of proportionality of odds highlighted. In this circumstance, the odds ratio may remain relevant (and probability value accurate) providing the treatment effect is relatively consistent across the spread of mRS scores.

*A.4.4.3.2 Alpha spending*

The Data Monitoring Committee performs safety assessments using the ‘3 standard error’ approach; hence, no significant spending of alpha will occur during the trial. All analyses will be two-tailed and a p-value of <0.05 will denote statistical significance; 95% confidence intervals will be provided. Adjustment for multiple comparisons will not be performed but all contrasts will be declared.

The primary and secondary outcomes follow here. Although not all the secondary outcomes will be presented in the primary paper, they will be used in secondary papers and are given here for completeness.

A.4.5 Primary outcome

A.4.5.1 Measure

The primary outcome is the 6-level ordered categorical measure of stroke frequency, and stroke severity (assessed using the mRS – see section 4.2.3) at day 90:

- Fatal stroke recurrence (mRS = 6) / recurrent stroke and mRS = 4 or 5 / recurrent stroke and mRS = 2 or 3 / recurrent stroke and mRS = 0 or 1 / TIA / no stroke or TIA

A.4.5.2 Analysis

Comparison between intensive and guideline antiplatelets of the outcome scale assessed using ordinal logistic regression (OLR) with adjustment for baseline covariates ([6](#_ENREF_6), [32](#_ENREF_32), [33](#_ENREF_33)).

A.4.5.3 Covariate adjustment

The covariates to be used in primary and secondary analyses will comprise:

- Index event (ischaemic stroke, TIA); Country; Guideline randomisation choice (aspirin/dipyridamole, clopidogrel, either)
- Age; Sex (female, male); Pre-morbid mRS; Time onset to randomisation; Number of antiplatelets before index event; Stroke syndrome (LACS, POCS, PACS, TACS); Systolic BP (SBP); Gastroprotection (yes, no); Use of heparin (yes, no); Stroke severity (National Institutes of Health Stroke Scale, NIHSS); Treated with rt-PA (yes, no); ABCD2 score; Number of TIA in last week.

Covariate adjustment will use the original (not dichotomised) data for ordered categorical (pre-morbid mRS, ABCD2, number of TIAs) and continuous (age, time, SBP, NIHSS) variables.

A.4.5.4 Subgroup analyses

The effect of the intervention (intensive antiplatelets versus guideline antiplatelets) on the primary outcome will be performed within the following pre-specified subgroups of participants. Subgroup analyses will be performed in those patients with a recurrent event using ordinal logistic regression. An interaction test between each of the following subgroups and treatment will be performed:

1. Geographical region: UK, other ¶
2. Age: <70 years, >70 years ¶
3. Sex: female, male
4. Index event: ischaemic stroke, transient ischaemic attack
5. Stroke/TIA syndrome: LACS, POCS, PACS, TACS ([2](#_ENREF_2)) ¶
6. National Institutes of Health Stroke Scale (NIHSS, stroke only): 0-3, >3 ¶
7. ABCD2 score (TIA only): 0-5, >5 ¶
8. Crescendo TIA (TIA only): No, Yes ¶
9. Number of antiplatelet agents at baseline: 0, 1, 2 ¶
10. Type of comparator: AD, C, either ¶
11. Systolic BP: <140, 141-160, >160 mmHg ¶
12. Time, event to randomisation >24, 12.1-24, <12 hours ¶
13. Use of low dose heparin: yes, no ¶
14. Treated with alteplase prior to randomisation (stroke only): yes, no
15. Gastroprotection: no, planned/on ¶
16. Carotid stenosis (ipsilateral >=50%) – no, yes ¶
17. Old lesion on baseline neuroimaging – no, yes ¶
18. Pre-morbid mRS: 0, >0

A.4.5.5 Sensitivity analyses

Several sensitivity analyses will be performed as a reference for the primary analysis. The methods of analysis are: BLR: binary logistic regression; CPHR: Cox proportional hazards regression; K-M: Kaplan-Meier curve; MLR: multiple linear regression; OLR: ordinal logistic regression

1. mRS in patients with recurrence, unadjusted OLR
2. mRS in all patients, adjusted and unadjusted OLR
3. Recurrent stroke or TIA, adjusted and unadjusted CPHR
4. Recurrent stroke, adjusted and unadjusted CPHR
5. Global outcome, mRS >1 and BI <95 in all patients, adjusted Wald ([34](#_ENREF_34), [35](#_ENREF_35))

Note: Comparison (d) is the usual analysis in trials assessing secondary stroke prevention.

A.4.6 Secondary outcomes

Secondary outcomes are listed by their timing. Analysis will be performed both adjusted (section 4.4.3) and unadjusted.

A.4.6.1 Day 7 ¶

1. Headache that required treatment or led to discontinuation BLR
2. Stroke recurrence/severity OLR
3. Composite vascular OLR
4. Venous thromboembolism OLR
5. Haemoglobin MLR

A.4.6.2 Day 35 (end of treatment)

1. Headache that required treatment or led to discontinuation BLR
2. Stroke recurrence/severity OLR
3. Myocardial infarction OLR
4. Composite vascular OLR
5. Acute coronary syndrome OLR
6. Venous thromboembolism OLR
7. Impairment (NIHSS, including death) MLR
8. Neurological deterioration BLR

A.4.6.3 Hospital discharge (collected at discharge or on death)

1. Length of stay in hospital MLR
2. Discharge disposition (death/institution/home) OLR

A.4.6.4 Day 90 (end of follow-up)

These are determined centrally by a telephone call between the patient (or carer) and an assessor blinded to treatment and earlier clinical information:

1. Myocardial infarction OLR
2. Composite vascular OLR
3. Venous thromboembolism OLR
4. Barthel Index (BI) MLR
5. Dead or disabled (BI <60) BLR
6. Quality of life/Health Utility Score (HUS, derived from EQ-5D) MLR
7. Quality of life (EQ-5D VAS) MLR
8. Telephone-Mini-Mental State Examination (t-MMSE) MLR
9. Telephone Interview Cognition Scale-Modified (TICS-M) MLR
10. Verbal fluency MLR
11. Zung Depression Scale (ZDS, mood) MLR
12. Disposition (death/institution/home) OLR

A.4.7 Safety analyses – Serious Adverse Events

Safety analyses are listed together with their intended method of analysis. All SAEs reported by the investigator are adjudicated by a member of the independent adjudicator panel who is blinded to treatment. The adjudicated SAE categorisation will be presented for all analyses. The number of participants with a SAE rather than the number of SAEs will be analysed. Comparisons will be performed on all patients.

A.4.7.1 Death

Time to death (censored at 110 days) CPHR

By cause Tabulation

A.4.7.2 Bleeding, day 7

1. Ordinal bleeding OLR

A.4.7.3 Bleeding, day 35 (end of treatment + 5 days of washout)

1. As in 4.6.2

A.4.7.4 Bleeding by day 90 (end of trial)

1. As in 4.6.2
2. Symptomatic intracranial haemorrhage CPHR/K-M
3. Major extracranial bleeding CPHR/K-M
4. Major bleeding (encompassing b+c) CPHR/K-M
5. Fatal bleeding CPHR/K-M
6. Major bleeding CPHR/K-M
7. Moderate bleeding CPHR/K-M
8. Mild bleeding CPHR/K-M
9. Any bleeding CPHR/K-M

A.4.7.5 SAEs, day 7

1. Serious adverse events OLR

A.4.7.6 SAEs by day 35 (end of treatment + 5 days of washout)

1. As in 4.6.5 OLR

A.4.7.7 SAEs by day 90 (end of follow-up)

1. As in 4.6.5 OLR
2. Time to serious adverse event CPHR/K-M
3. Tabulation by type

Serious adverse events by 90 days will be tabulated as the number of participants with an event in each treatment group, and the number of events in each treatment group, by the following subgroups.

1. System organ class
2. Event category within system organ class
3. Time of onset relative to treatment (before, during, after)
4. Median time to event by event category
5. Severity (mild, moderate, severe)
6. Relationship to study drug (definitely not, unlikely, possibly, probably, definitely)
7. Fatal, non-fatal
8. Fatal by event category

Common serious adverse events that will be reported are listed in Appendix C.6, table 6.

A.4.8 Compliance

Compliance with allocated treatment will be tabulated:

1. Number (%) of participants who received at least the first allocated treatment
2. Number (%) of participants who received at least the first 7 days of allocated treatment
3. Number (%) of participants who received at least the first 21 days of allocated treatment
4. Number (%) of participants who received all of their allocated treatment
5. Number (%) of participants who did not receive any of the allocated treatment

In those participants who did not receive all of the first 7 days of allocated treatment, the reason for non-compliance will be given:

1. Discharged before day 7
2. Adverse event, unacceptable
   1. Headache
   2. Bleeding
3. Physician withdrew participant. [This will be recorded as an unacceptable adverse event if withdrawal due to headache or bleeding.]
4. Serious adverse event
5. Consent withdrawn by participant or relative. [This will be recorded as an unacceptable adverse event if withdrawal due to headache or bleeding.]
6. Death
7. Other reason

Note: If not admitted, or discharged before day 28-30, patients are given the remaining part of their course of antiplatelet(s) to complete out of hospital.

A.4.9 Analysis methods

Data variables will, in the main, be analysed using binary logistic regression (BLR), Cox proportional hazards regression (CPHR); ordinal logistic regression (OLR) or multiple logistic regression (MLR), thereby allowing adjustment for baseline measures and other covariates (as listed partially in section 4.4.3).

A.4.9.1 Clinical variables

- BLR: Death; Death or deterioration (day 7); Recurrence (day 7); Symptomatic intracranial bleed (sICH); Discharged to or at Home
- CPHR: Death; bleeding; serious adverse events
- OLR: Recurrence by severity (using mRS); MI, Vascular; VTE; discharge disposition
- MLR: NIHSS; Length of stay in hospital; BI; t-MMSE; TICS; Animal naming; ZDS; HUS; EQ-VAS

A.4.9.2 Imaging variables (ICH measures refer to any scan after randomisation)

- BLR: Loss of grey-white definition; Loss of basal ganglia outline; Hypodensity; Mass effect; Second lesion; Hyperdense artery
- OLR: Lesion site; Lesion size: MCA, lacunar, cerebellum or brainstem; Mass effect, degree [/6]; Haemorrhagic Transformation of Infarct; Hypoattenuation; Mass effect score (greatest of Uncal> shift> ventricular> sulcal); Middle Cerebral Artery 1/3 (if MCA, >1/3, <1/3, no lesion); ASPECTS score; ICH diameter, max – adjudicated; ICH shape [/5]; ICH density [/5]; IVH Graeb [/12];([36](#_ENREF_36)) IVH modified Graeb [/32];([37](#_ENREF_37))
- MLR: Volume (ABC/2), absolute (ml); Volume (ABC/2), relative (%); Diameter, max (cm); Haematoma shape index; Haematoma mean density; Haematoma density index; IVH volume

A.4.9.3 Procedures

The primary paper will be analysed using two separate sets of statistical programmes, one written specifically for the purpose and the other based on the Data Monitoring Committee report. A further analysis of a sub-set of the main results will be performed using a third statistical programme. This approach will allow the results to be cross-checked.

APPENDIX B. DEFINITIONS

B.1 DEFINITIONS OF EVENTS/OUTCOMES

Stroke, myocardial infarction and bleeding (major, moderate) are adjudicated independently by two adjudicators; if they differ in type of vascular event or severity of bleeding, a third adjudicator also assesses the event and a majority view is recorded for analysis. Serious adverse events are assessed by a single adjudicator. All adjudicators are blinded to treatment assignment

B.1.1 Asymptomatic Intracerebral Haemorrhage (aICH) ¶

Any haemorrhage seen on CT/MRI scanning, if done after randomisation, with no neurological deterioration (as defined below).

B.1.2 Bleeding

B.1.2.1 Major bleed ([38](#_ENREF_38))

- - Fatal bleeding, and/or
  - Symptomatic bleeding in a critical area or organ, such as intracranial, intraspinal, intraocular, retroperitoneal, intraarticular or pericardial, or intramuscular with compartment syndrome, and/or
  - Bleeding causing fall in haemoglobin of 20 g/l (1.24 mmol/l) or more, or leading to transfusion of 2 or more units of whole blood or red cells.

B.1.2.2 Moderate bleed

- Not major bleed, and
- Bleeding causing fall in haemoglobin of greater than 10 g/l (0.62 mmol/l) but less than 20 g/l (1.24 mmol/l) and leading to no transfusion, or transfusion of only 1 unit of whole blood or red cells.

B.1.2.3 Minor Bleed

- Not major or moderate bleed, and
- Comprising bruising, ecchymoses, gingival bleed or similar other type of bleeding.

Note: Excludes asymptomatic ICH

B.1.3 Deep vein thrombosis (DVT), symptomatic

The clinical suspicion of DVT will need confirmation by either venography or ultrasound examination.

B.1.4 Disposition

Disposition is categorised as death, institution or home:

- Institution refers to warden controlled, residential home, care home, nursing home, still an inpatient, or readmitted to hospital.
- Home refers to home alone, home with spouse/carer, or at carer’s home.

B.1.5 Extracranial haemorrhage, major

An extracranial bleed that is major in severity (see 1.1).

B.1.6 Feeding status

Feeding status is defined as:

- Oral: normal diet, soft diet
- Non-oral: nasogastric tube fed, PEG-tube fed, iv/sc fluids, no feeding/fluids

B.1.7 Headache, requiring treatment or cessation of treatment

A headache occurring during treatment that necessitates intervention, including withdrawing antiplatelet treatment.

B.1.8 Intracerebral haemorrhage, secondary on CT/MRI scanning

1. *Haemorrhagic Infarct (HI):* petechial infarction without space occupying effect
2. HI1 – small petechiae
3. HI2 – more confluent petechiae
4. *Parenchymal Haemorrhage (PH):* haemorrhage with mass effect
   1. PH1-<30% of the infarcted area with mild space occupying effect
   2. PH2->30% of the infarcted area with significant space occupying effect

Note: Patients with PH should not be enrolled into the trial.

B.1.9 Intracerebral haemorrhage, symptomatic (sICH)

Neurological deterioration or death, associated with intracerebral haemorrhage found on CT/MR scan or autopsy. The haemorrhage must be the predominant cause of the neurological deterioration.

Note: This excludes other forms of intracranial haemorrhage, including extra-dural, sub-dural and subarachnoid haemorrhage, which will be reported separately.

B.1.10 Intracranial haemorrhage

Symptomatic ICH, extra-dural haemorrhage, sub-dural haemorrhage, and/or subarachnoid haemorrhage.

B.1.11 Myocardial infarction (MI)

Either one of the following criteria satisfies the diagnosis for an acute, evolving or recent MI:([39](#_ENREF_39))

- Typical rise and fall of biochemical markers of myocardial necrosis with at least two of the following criteria:
  - Ischaemic symptoms;
  - Development of pathologic Q waves on the ECG;
  - ECG changes indicative of ischemia (ST segment elevation or depression); or
  - Coronary artery intervention (e.g., coronary angioplasty).
- Pathological findings of an acute MI.

STEMI – MI with ST elevation on ECG

NSTEMI - MI with no ST elevation on ECG

B.1.12 Neurological deterioration

An increase in NIHSS by 4 points or more over the baseline value.

B.1.13 Pulmonary embolism (PE), symptomatic

The clinical suspicion of PE will need confirmation by either high-probability ventilation-perfusion lung scintigraphy, pulmonary angiography, inconclusive V/Q scan and DVT, or lead to death.

B.1.14 Recurrent stroke, symptomatic

A stroke, defined as below, occurring after the qualifying stroke, or a progression of neurological symptoms or signs (increase in NIHSS score >4) in the same vascular territory as the index event. Classified as haemorrhagic or ischaemic (if documented by CT/MR scan or autopsy), or of unknown type. The time from stroke onset and lesion side will be noted.

Note: This definition deliberately does not attempt to differentiate true recurrence from extension of the presenting lesion since this is clinically and radiologically difficult unless recurrence occurs in a new arterial territory.

B.1.15 Stroke

A clinical syndrome characterised by rapidly developing clinical symptoms and/or signs of focal (and at times global) loss of cerebral function with symptoms lasting ≥ 24 hours or leading to death, with no apparent cause other than that of vascular origin.

B.1.16 Transient ischaemic attack (TIA)

A sudden focal neurological deficit of the brain or eye, presumed to be of vascular origin and lasting less than 24 hours.

Note: The tissue diagnosis of TIA based on the results of magnetic resonance imaging will not be used since MRI is not routinely available out of hours at many participating hospitals.

B.1.17 Time at home

Calculated as time from date of discharge to day 90 or death if earlier; those who die in hospital or are discharged to a non-home setting are given a score of zero. Readmission to hospital is not counted in this time.

B.1.18 Unstable angina

Presence of acute cardiac chest pain at rest without ST elevation on ECG and elevation in cardiac enzymes.

B.1.19 Venous thromboembolism, symptomatic

Symptomatic DVT and/or symptomatic pulmonary embolism

B.2 OTHER DEFINITIONS AND NOTES

B.2.1 Geographic regions

This will be categorised depending on which countries participate in the trial. As of 2014, this will include Europe/non-UK, UK, and Other.

B.2.2 Haemodynamic variables, calculated

These are calculated from systolic blood pressure (SBP), diastolic blood pressure (DBP) and heart rate (HR) measurements at baseline (day 0) and on days 1-6. Mean, peak, and standard deviation (SD), of SBP/DBP/HR are calculated from available measurements at that time point.

Coefficient of Variation, CV=SD/mean.

1. SBP, mean
2. SBP, peak
3. SBP, intra-visit variability = SD of SBP separately at each time point
4. SBP, CV
5. DBP, mean
6. DBP, peak
7. DBP, intra-visit variability = SD of DBP separately at each time point
8. DBP, CV
9. HR, mean
10. HR, peak
11. HR, intra-visit variability = SD of HR separately at each time point
12. HR, CV
13. Mean arterial pressure (MAP) = mean DBP + (mean SBP / 3)
14. Mean pressure (MP) = (mean DBP + mean SBP) / 2
15. Pulse pressure (PP) = mean SBP – mean DBP
16. Pulse pressure index (PPI) = PP / MAP
17. Rate-pressure product (RPP) = mean SBP x mean HR
18. Mean SBP on days 1 and 7
19. Inter-visit Variability (SBP-inter SD) = SD of SBP on days 1 and 7
20. CV SBP on days 1 and 7
21. SBP peak on days 1 and 7

B.2.3 Acute Stroke Unit (ASU)

A high-dependency nursing unit (or area) caring only/mainly for patients with acute stroke and providing close monitoring of neurological and vascular signs.

B.2.4 Stroke Rehabilitation Unit (SRU)

A dedicated rehabilitation unit (or area) caring only/mainly for patients with recent stroke and providing multi-disciplinary therapy (e.g. physiotherapy, occupational therapy, speech & language therapy).

B.2.5 Final diagnosis

The diagnosis given at the time of randomisation, i.e. ischaemic stroke versus TIA.

B.2.6 Date of death

Dates of death have multiple sources and these are ranked for determining the date (in case a particular source is missing):

1. MRIS/ONS feedback, if UK patient
2. Serious adverse event form
3. Day 7 form
4. Day 35 form
5. Hospital discharge form

If there is a discrepancy in the recorded date then the site are approached to confirm the correct date.

B.2.7 EuroQol-5D (EQ-5D) and Health Utility States (HUS)

EQ-5D is a measure of health status and is composed of the EQ-5D descriptive system and EQ visual analogue scale (EQ VAS). The EQ VAS is a self-rated scale from 0 (worst imaginable health state) to 100 (best imaginable health state). The EQ-5D descriptive system has 5 dimensions: mobility, self-care, usual activities, pain/discomfort and anxiety/depression. Each dimension has 3 levels; no problems (level 1), some problems (level 2), severe problems (level 3).([40](#_ENREF_40))

There are 243 possible health states. Each health state is referred to by a 5 digit code, e.g., 11111. The EQ-5D health states can be converted into a single summary index ranging from 1.00, i.e. full health (11111), to -0.594 (33333, worst possible state using the UK version of the time trade-off algorithm), with death assigned to 0.([40](#_ENREF_40)) The conversion is made by applying the formula in Table 5, which deducts weights applied to each dimension from the starting value of 1. The UK version of the EQ-5D index algorithm using the time trade off value set will be used for all patients.([31](#_ENREF_31))

EQ-5D index scoring algorithm (Time trade-off: UK version)

|  | Weight |
| --- | --- |
| Full health (11111) | 1 |
| At least one 2 or 3 | -0.081 |
| At least one 3 | -0.269 |
| Mobility |  |
| Score 2 | -0.069 |
| Score 3 | -0.314 |
| Self-care |  |
| Score 2 | -0.104 |
| Score 3 | -0.214 |
| Usual activities |  |
| Score 2 | -0.036 |
| Score 3 | -0.094 |
| Pain/discomfort |  |
| Score 2 | -0.123 |
| Score 3 | -0.386 |
| Anxiety/depression |  |
| Score 2 | -0.071 |
| Score 3 | -0.236 |

For example, a health state of 32211 will convert to 0.196 (=1-0.81-0.269-0.314-0.104-0.071).

B.2.8 Protocol violations

Patients who have one or more of the following protocol violations will be excluded from the *per protocol* population (see protocol V1.5):

- Randomisation > 48 hours from onset of symptoms
- Age <50 years
- For ischaemic stroke:
  - No cranial imaging results available prior to randomisation
  - Isolated sensory symptoms, vertigo, dizziness, or facial weakness as presenting symptoms of the index event
- For TIAs:
  - Limb weakness and/or dysphasia lasting <10 minutes
  - ABCD2 score <4 and not a crescendo TIA and not on dual antiplatelet therapy
- Failure to obtain appropriate consent prior to randomisation
- Known premorbid dependency (mRS) >2
- Participant unable to swallow and does not have enteral access
- Baseline cranial imaging shows parenchymal haemorrhagic transformation (PH I/II)
- Subarachnoid haemorrhage at baseline, prior to randomisation
- Intracerebral haemorrhage at baseline, prior to randomisation
- On anticoagulation therapy except low dose low molecular weight heparin (for prophylaxis)
- Participant has taken dipyridamole between the index event and prior to stroke randomisation, where clopidogrel is the control treatment
- Participant has taken clopidogrel between the index event and prior to stroke randomisation, where combined aspirin and dipyridamole is the control treatment
- Thrombolysis less than 24 hours prior to randomisation
- Presumed cardioembolic stroke or history of atrial fibrillation
- Concomitant STEMI or NSTEMI at baseline, prior to randomisation
- Baseline SBP reading >185 mmHg or DBP >110 mmHg
- Major bleeding within one year prior to randomisation
- Planned surgery within the 3-month follow-up period
- Randomising event was secondary to a surgical procedure
- Glasgow Coma Score < 8 at baseline, prior to randomisation
- Known history of dementia at baseline, prior to randomisation
- Known probable life expectancy of less than 6 months
- Unavailable for follow-ups
- Female patient of childbearing potential, pregnant or breastfeeding at randomisation
- Patient receiving treatment that they are not randomised to
- Patients who do not have a post thrombolysis scan
- Patient does not receive the correct loading dose of randomised treatment

B.2.9 CT/MR image adjudication (as per IST-3/ENOS scheme ©JMWardlaw 2012 ([20](#_ENREF_20), [41](#_ENREF_41)))

A pre-randomisation CT or MRI brain scan is mandatory in patients enrolled with an ischaemic stroke, and optional in those with a TIA; these scans are assessed by an independent adjudicator blinded to treatment assignment. Any post-randomisation scan performed for clinical reasons will also be adjudicated. Lesion size is assessed by an independent assessor, i.e. not by the investigator.

B.2.9.1 Infarct visibility

Infarct size is coded (small=1 through to large=4) using adjudicated data for both baseline and, where done, day 7 CT/MR scans.

B.2.9.2 Site Condensed code

M† =MCA = any lesion in the MCA territory see 2.9.3 below

AS =Infarct of up to half of ACA territory 1

AL =Infarct of more than half of ACA territory 2

PS =Infarct of up to half of PCA territory 1

PL =Infarct of more than half of PCA territory 3

MAS=M+AS† 3 if MCA 1 or 2; 4 if MCA 3 or 4

MAL=M+AL† 3 if MCA 1 or 2; 4 if MCA 3 or 4

MPS=M+PS† 3 if MCA 1 or 2; 4 if MCA 3 or 4

MPL=M+PL† 3 if MCA 1 or 2; 4 if MCA 3 or 4

MAP=Infarct of whole MCA, ACA and PCA territories 4

L† =Lacunar 1

B† =Borderzone 1

C† =Cerebellum see 2.9.3 below

S† =Brainstem see 2.9.3 below

CS† =Cerebellum and brainstem 3

† code sub-territory sites as in 2.9.3

B.2.9.3 Sub-territory sites

MCA sub-territory codes

1=small cortical infarct 1

2=basal ganglia infarct (>2x2x2cm) 2

3=infarct of white matter lateral to lateral ventricle (>2x2x2cm) 2

4=infarct of anterior half of peripheral MCA territory 2

5=infarct of posterior half of peripheral MCA territory 3

6=infarct of whole of peripheral MCA territory 3

7=6+infarct of lateral part of basal ganglia 4

8=infarct of whole of MCA territory 4

Lacunar/Borderzone sub-territory codes all 1

9=lacune in internal capsule/lentiform

10=lacune in internal border zone

11=lacune in centrum semiovale

12=lacune in thalamus

13=lacune in brainstem, including pons (not shown)

14=anterior (mainly) border zone

15=posterior (mainly) border zone

Cerebellum sub-territory codes

16=small cortical (not shown) 1

17=<1/2 hemisphere (medium) (not shown) 2

18=>1/2 hemisphere (not shown) 3

Brainstem sub-territory codes

19=small, i.e.<1/2 medulla (not shown) 1

20=extensive, i.e. pons + medulla (not shown) 2

Degree of mass effect codes

0=no swelling

1=effacement of sulci overlying stroke

2=1+minor effacement of adjacent lateral ventricle

3=1+complete effacement of adjacent lateral ventricle

4=1+effacement of lateral and third ventricle

5=4+shift of the midline away from side of ventricle

6=5+effacement of basal cisterns

B.2.9.4 Infarcts

Loss of grey/white matter cortex definition yes/no

Loss of basal ganglia outline yes/no

Hypodensity present yes/no

Mass effect yes/no, if yes each yes/no

- Succal effacement
- Ventricular effacement
- Midline shift
- Uncal herniation

1/3 MCA territory yes/no

ASPECT score, for each region yes/no

Second discrete lesion yes/no

Hyperdense artery yes/no, if yes then site

B.2.9.5 Haemorrhages

By presence, importance and size (<3, 3-5, 5-8, >8 cm):

- Petechial
- Haemorrhagic transformation of infarct
- Parenchymal haematoma (no infarct)
- Parenchymal haematoma (remote from infarct)
- Subdural haematoma
- Subarachnoid haemorrhage
- Extradural haemorrhage
- Bleeding AVM

B.2.9.6 Other features

Reduction in brain tissue volume: none, moderate severe:

- Central
- Cortical

Periventricular lucencies: none, restricted to region adjoining ventricles, from ventricle to cortex

- Anterior white matter
- Posterior white matter

Old vascular lesions: each yes/no

- Cortical infarct(s)
- Striatocapsular infarct(s)
- Borderzone infarct(s)
- Lacunar infarct(s)
- Brainstem/cerebellar infarct(s)
- Previous intracranial haemorrhage
- Presence and number of microhaemorrhages (if appropriate MRI sequences available)

Non-stroke lesions, each yes/no

- Cerebral tumour
- Encephalitis
- Cerebral abscess
- Other, e.g. contusion

APPENDIX C. PRIMARY PUBLICATION / NIHR HTA PUBLICATION - TABLES AND FIGURES

Authors

TARDIS Trial Investigators

C.1. Baseline characteristics

Table 1. Baseline characteristics of patients enrolled into the TARDIS trial, by treatment group. Data are number (%), median [interquartile range] or mean (standard deviation).

|  | N | IS | TIA | Intensive  ACD | Guideline  AD and C | AD | C |
| --- | --- | --- | --- | --- | --- | --- | --- |
| Number of patients |  |  |  |  |  |  |  |
| Age (years) † |  |  |  |  |  |  |  |
| Sex, male (%) † |  |  |  |  |  |  |  |
| Geographical region ‡ |  |  |  |  |  |  |  |
| Europe, rest of |  |  |  |  |  |  |  |
| UK |  |  |  |  |  |  |  |
| World, rest of |  |  |  |  |  |  |  |
| Pre-stroke mRS [/6] |  |  |  |  |  |  |  |
| 0 (%) |  |  |  |  |  |  |  |
| 1-2 |  |  |  |  |  |  |  |
| Medical history (%) |  |  |  |  |  |  |  |
| Hypertension, treated ‡ |  |  |  |  |  |  |  |
| Hyperlipidaemia, treated |  |  |  |  |  |  |  |
| Antiplatelet agent |  |  |  |  |  |  |  |
| Aspirin |  |  |  |  |  |  |  |
| Aspirin & dipyridamole |  |  |  |  |  |  |  |
| Clopidogrel |  |  |  |  |  |  |  |
| Other |  |  |  |  |  |  |  |
| Atrial fibrillation, current/previous |  |  |  |  |  |  |  |
| Stroke |  |  |  |  |  |  |  |
| Ischaemic heart disease |  |  |  |  |  |  |  |
| Peripheral arterial disease |  |  |  |  |  |  |  |
| Smoking, current (%) |  |  |  |  |  |  |  |
| Qualifying event (%) † |  |  |  |  |  |  |  |
| Ischaemic stroke |  |  |  |  |  |  |  |
| Transient Ischaemic Attack |  |  |  |  |  |  |  |
| Crescendo |  |  |  |  |  |  |  |
| Dual antiplatelets |  |  |  |  |  |  |  |
| Non-stroke |  |  |  |  |  |  |  |
| Side of lesion, right (%) |  |  |  |  |  |  |  |
| Weakness (%) |  |  |  |  |  |  |  |
| Sensory loss (%) |  |  |  |  |  |  |  |
| Dysphasia (%) |  |  |  |  |  |  |  |
| Isolated |  |  |  |  |  |  |  |
| Neglect (%) |  |  |  |  |  |  |  |
| Hemianopia (%) |  |  |  |  |  |  |  |
| Isolated |  |  |  |  |  |  |  |
| NIHSS (/42) † |  |  |  |  |  |  |  |
| ABCD2 score [/7] ∆ |  |  |  |  |  |  |  |
| Glasgow Coma Scale [/15] |  |  |  |  |  |  |  |
| OCSP classification (%) |  |  |  |  |  |  |  |
| Total anterior † |  |  |  |  |  |  |  |
| Partial anterior |  |  |  |  |  |  |  |
| Lacunar |  |  |  |  |  |  |  |
| Posterior |  |  |  |  |  |  |  |
| TOAST classification (%) Ω∫ |  |  |  |  |  |  |  |
| Cardioembolic |  |  |  |  |  |  |  |
| Large vessel |  |  |  |  |  |  |  |
| Small vessel |  |  |  |  |  |  |  |
| Mixed |  |  |  |  |  |  |  |
| Other |  |  |  |  |  |  |  |
| Haemodynamics |  |  |  |  |  |  |  |
| Systolic BP (mmHg) † |  |  |  |  |  |  |  |
| Diastolic BP (mmHg) |  |  |  |  |  |  |  |
| Heart rate, mean (bpm) |  |  |  |  |  |  |  |
| Brain imaging (%) |  |  |  |  |  |  |  |
| Normal/no lesion |  |  |  |  |  |  |  |
| Ischaemic stroke |  |  |  |  |  |  |  |
| Non stroke |  |  |  |  |  |  |  |
| No brain scan |  |  |  |  |  |  |  |
| Time onset to randomisation [hours] † |  |  |  |  |  |  |  |
| Index event |  |  |  |  |  |  |  |
| Ischaemic stroke |  |  |  |  |  |  |  |
| TIA |  |  |  |  |  |  |  |
| Time (%) |  |  |  |  |  |  |  |
| <12 |  |  |  |  |  |  |  |
| 13-24 |  |  |  |  |  |  |  |
| >24 |  |  |  |  |  |  |  |
| Thrombolysis † |  |  |  |  |  |  |  |
| Time to thrombolysis [hours] |  |  |  |  |  |  |  |

† Minimisation variable; ‡ Stratification variable; Ω Ischaemic patients only; ∆ Protocol violation

bpm: beats per minute; NIHSS: National Institutes of Health Stroke Scale; mRS: modified Rankin Scale; OCSP: Oxford Community Stroke Project TOAST: Trial of ORG 10172 in Acute Stroke Treatment

C.2. Adherence

Table 2. Adherence and reasons for non-adherence to randomised treatment, by treatment group. Patients receiving at least first 21 doses are considered to have had full treatment. Data are number (%). Comparison of total withdrawals by binary logistic regression with 95% confidence intervals.

| Reasons for non-compliance |  | N | All | Intensive  ACD | Guideline  all | AD | C | 2p |  |
| --- | --- | --- | --- | --- | --- | --- | --- | --- | --- |
| Participants randomised |  |  |  |  |  |  |  |  |  |
| Compliance | |  |  |  |  |  |  |  |  |
| Received first treatment | |  |  |  |  |  |  |  |  |
| Received first 7 days of treatment | |  |  |  |  |  |  |  |  |
| Received >=28 days of treatment | |  |  |  |  |  |  |  |  |
| Received some treatments | |  |  |  |  |  |  |  |  |
| Did not receive any randomised treatment | |  |  |  |  |  |  |  |  |
| Number of tablets taken | |  |  |  |  |  |  |  |  |
| Mean (SD) | |  |  |  |  |  |  |  |  |
| Median [IQR] | |  |  |  |  |  |  |  |  |
| Mode | |  |  |  |  |  |  |  |  |
| Non-compliance by day 7 (%) | |  |  |  |  |  |  |  |  |
| Discharged |  |  |  |  |  |  |  |  |  |
| Withdrew |  |  |  |  |  |  |  |  |  |
| Adverse event, unacceptable |  |  |  |  |  |  |  |  |  |
| Headache |  |  |  |  |  |  |  |  |  |
| Bleeding |  |  |  |  |  |  |  |  |  |
| Serious adverse event |  |  |  |  |  |  |  |  |  |
| Death |  |  |  |  |  |  |  |  |  |
| Other |  |  |  |  |  |  |  |  |  |

Odds ratio= , 95% confidence interval = - , 2p= .

Note: When discharged, patients were given the remaining part of their course of antiplatelet(s) to complete at home.

C.3. Outcomes

Table 3. Primary and secondary outcomes at day 90 in patients who had a stroke or TIA, by treatment group. Stroke/TIA is given by severity; where a patient had more than one event, the most severe event is used. Data are number (%), median [interquartile quartile range] or mean (standard deviation). Comparison by binary logistic regression, ordinal logistic regression, or multiple linear regression, shown as odds ratio (OR) median [interquartile range] or mean difference (MD), with 95% confidence intervals. Analyses are adjusted unless stated.

| Outcome | N | Intensive | Guideline | OR/MD (95% CI)  adjusted | P | OR/MD (95% CI)  unadjusted | P |
| --- | --- | --- | --- | --- | --- | --- | --- |
| Patients | N | N1 | N2 | - |  |  |  |
|  |  |  |  |  |  |  |  |
| Primary outcome, day 90 |  |  |  |  |  |  |  |
| Patients with outcome | N | N1 | N2 |  |  |  |  |
| Ordinal stroke/TIA [/6] |  |  |  | OLR OR |  |  |  |
| using mRS |  |  |  |  |  |  |  |
| 6 death |  |  |  | HR OR |  |  |  |
| 4, 5 |  |  |  |  |  |  |  |
| 2, 3 |  |  |  |  |  |  |  |
| 0, 1 |  |  |  |  |  |  |  |
| TIA |  |  |  | BLR OR |  |  |  |
| No stroke/TIA |  |  |  |  |  |  |  |
| Stroke or TIA (%) |  |  |  | HR OR |  |  |  |
| Stroke (%) |  |  |  | HR OR |  |  |  |
| Ischaemic |  |  |  | HR OR |  |  |  |
| Haemorrhagic |  |  |  | HR OR |  |  |  |
| Unknown |  |  |  | HR OR |  |  |  |
| mRS>2 |  |  |  | HR OR |  |  |  |
|  |  |  |  |  |  |  |  |
| Secondary outcomes, day 90 |  |  |  |  |  |  |  |
| Barthel Index (/100) ‡ |  |  |  | MR MD |  |  |  |
| BI <60 (%)‡ |  |  |  | BLR OR |  |  |  |
| ZDS (/102.5) ‡ |  |  |  | MR MD |  |  |  |
| t-MMSE (/18) ‡ |  |  |  | MR MD |  |  |  |
| TICS-M (/37)‡ |  |  |  | MR MD |  |  |  |
| Animal naming |  |  |  | MR MD |  |  |  |
| EQ-VAS (/100) ‡ |  |  |  | MR MD |  |  |  |
| EQ-5D HUS (/1) ‡ |  |  |  | MR MD |  |  |  |

BI: Barthel Index; EQ: EuroQol; EQ-5D3: EQ-5 dimensions 3 levels; EQ-VAS: EQ-Visual Analogue Scale; HUS (from EQ-5D3): Health Utility Status; mRS: modified Rankin Scale; t-MMSE: t-Mini-Mental State Examination; TICS-M: Telephone Interview Cognition Scale-M; ZDS: Zung Depression Scale

‡: Death = BI -5, t-MMSE -1, TICS-M -1, EQ-VAS -1, HUS 0, mRS 6, ZDS 102.5

C.4. Outcomes in all patients

Table 4. Secondary outcomes in all patients, by treatment group. Data are number (%), median [interquartile quartile range] or mean (standard deviation). Comparison by binary logistic regression, ordinal logistic regression, or multiple linear regression, shown as odds ratio (OR) median [interquartile range] or mean difference (MD), with 95% confidence intervals. Analyses are adjusted unless stated.

| Outcome | | N | Intensive | Guideline | OR/MD (95% CI), adjusted | 2p |
| --- | --- | --- | --- | --- | --- | --- |
| Patients |  |  |  |  |  |  |
|  |  |  |  |  |  |  |
| Day 7 | |  |  |  |  |  |
| Death (%) | |  |  |  | BLR OR |  |
| Recurrent stroke or TIA (%) | |  |  |  | BLR OR |  |
| Impairment (NIHSS) (/42) ‡ | |  |  |  | MR MD |  |
| Neuro. deterioration (%) ƒ | |  |  |  | BLR OR |  |
| Headache (%) | |  |  |  | BLR OR |  |
|  | |  |  |  |  |  |
| Day 35 | |  |  |  |  |  |
| Death (%) | |  |  |  | BLR OR |  |
| Recurrent stroke or TIA (%) | |  |  |  | BLR OR |  |
| Impairment (NIHSS) (/42) ‡ | |  |  |  | MR MD |  |
| Neuro. deterioration (%) ƒ | |  |  |  | BLR OR |  |
| Headache (%) | |  |  |  | BLR OR |  |
| Venous thromboembolism | |  |  |  | HR OR |  |
| Fatal PE | |  |  |  | OLR OR |  |
| PE | |  |  |  |  |  |
| DVT | |  |  |  |  |  |
| None | |  |  |  |  |  |
|  | |  |  |  |  |  |
| Hospital discharge events | |  |  |  |  |  |
| Hospital stay (days) | |  |  |  | MR MD |  |
| Discharge disposition (%) | |  |  |  | OLR OR |  |
|  | |  |  |  |  |  |
| Day 90 ‡ | |  |  |  |  |  |
| Death (%) | |  |  |  | HR OR |  |
| Vascular death | |  |  |  | HR OR |  |
| Myocardial infarction (%) | |  |  |  | HR OR |  |
| Fatal | |  |  |  | OLR OR |  |
| MI | |  |  |  | - |  |
| Angina, unstable | |  |  |  | - |  |
| Angina, stable | |  |  |  | - |  |
| No MI/UA | |  |  |  | - |  |
| modified Rankin Scale | |  |  |  | OLR OR |  |
| Barthel Index (/100) | |  |  |  | MR MD |  |
| Barthel Index <60 (%) | |  |  |  | BLR OR |  |
| Zung Depression Scale (/100) | |  |  |  | MR MD |  |
| t-MMSE (/18) | |  |  |  | MR MD |  |
| TICS-M (/37) | |  |  |  | MR MD |  |
| EQ-VAS (/100) | |  |  |  | MR MD |  |
| EQ-5D3 HUS (/1) | |  |  |  | MR MD |  |
| Adverse events (%) | |  |  |  | BLR OR |  |
| Fatal | |  |  |  | OLR OR |  |
| Serious | |  |  |  | - |  |
| Adverse | |  |  |  | - |  |
| None | |  |  |  | - |  |

BI: Barthel Index; EQ: EuroQoL; EQ-5D3: EQ-5 dimensions 3 levels; EQ-VAS: EQ-Visual Analogue Scale; HUS (from EQ-5D3): Health Utility Status; mRS: modified Rankin Scale; Neuro. deterioration: Neurological deterioration; t-MMSE: t-Mini-Mental State Examination; TICS-M: Telephone Interview Cognition Scale-M; ZDS: Zung Depression Scale

† Ordinal stroke/TIA recurrence: fatal / mRS 3-5 / mRS 0-2 / TIA / no stroke-TIA

ƒ Neurological deterioration: decrease in NIHSS >3 points

‡: Death = BI -5, t-MMSE -1, TICS-M -1, EQ-VAS -1, HUS 0, mRS 6, NIHSS 43, ZDS 102.5

C.5. Bleeding

Table 5. Cumulative bleeding at 7, 35 and 90 days, by treatment group. Bleeding is given by severity. Where a patient had more than one bleed, the most severe bleed is used at each time point; at day 90 the severity of the first bleed is also given. Data are number (%), median [interquartile quartile range] or mean (standard deviation). Comparison by binary logistic regression, ordinal logistic regression, or multiple linear regression, shown as odds ratio (OR) median [interquartile range] or mean difference (MD), with 95% confidence intervals. Analyses are adjusted unless stated.

| Outcome | N | Intensive | Guideline | OR/MD (95% CI), adjusted | 2p |  |
| --- | --- | --- | --- | --- | --- | --- |
| Patients |  |  |  |  |  |  |
|  |  |  |  |  |  |  |
| Day 7 (or discharge) |  |  |  |  |  |  |
| Bleeding, most severe (%) | |  |  | BLR OR |  |  |
| Fatal ([38](#_ENREF_38)) | |  |  | OLR OR |  |  |
| Major | |  |  | - |  |  |
| Moderate | |  |  | - |  |  |
| Mild | |  |  | - |  |  |
| None | |  |  | - |  |  |
| sICH (%) | |  |  | BLR OR |  |  |
| mECB (%) | |  |  | BLR OR |  |  |
| Haemoglobin (g.dl^-1^) | |  |  | MLR MD |  |  |
|  | |  |  |  |  |  |
| Day 35 |  |  |  |  |  |  |
| Bleeding, most severe (%) |  |  |  | BLR OR |  |  |
| Fatal ([38](#_ENREF_38)) |  |  |  | OLR OR |  |  |
| Major |  |  |  | - |  |  |
| Moderate |  |  |  | - |  |  |
| Mild |  |  |  | - |  |  |
| None |  |  |  | - |  |  |
| sICH (%) |  |  |  | BLR OR |  |  |
| mECB (%) |  |  |  | BLR OR |  |  |
| Haemoglobin (g.dl^-1^) | |  |  | MLR MD |  |  |
|  |  |  |  |  |  |  |
| Day 90 |  |  |  |  |  |  |
| Bleeding, most severe (%) |  |  |  | BLR OR |  |  |
| Fatal ([38](#_ENREF_38)) |  |  |  | OLR OR |  |  |
| Major |  |  |  | - |  |  |
| Moderate |  |  |  | - |  |  |
| Mild |  |  |  | - |  |  |
| None |  |  |  | - |  |  |
| sICH (%) |  |  |  | BLR OR |  |  |
| mECB (%) |  |  |  | BLR OR |  |  |
|  |  |  |  |  |  |  |
| Day 90 |  |  |  |  |  |  |
| Bleeding, first (%) |  |  |  | BLR OR |  |  |
| Fatal ([38](#_ENREF_38)) |  |  |  | OLR OR |  |  |
| Major |  |  |  | - |  |  |
| Moderate |  |  |  | - |  |  |
| Mild |  |  |  | - |  |  |
| None |  |  |  | - |  |  |

mECB: major extracranial bleed; sICH: symptomatic intracranial haemorrhage

C.6. Serious adverse events

Table 6. Cumulative number of patients with at least one serious adverse event by day 35 (end of treatment washout), and day 90 (end of follow-up), by treatment group. Data are number (%) of affected patients. Comparison by binary logistic regression.

| Number (%) | Time to event (days) | All |  |  | Fatal |  |  |
| --- | --- | --- | --- | --- | --- | --- | --- |
| Cause | Median | Intensive | Guideline | P | Intensive | Guideline | P |
| By day 7 | - |  |  |  |  |  |  |
| By day 35 | - |  |  |  |  |  |  |
| By day 90 | - |  |  |  |  |  |  |
|  |  |  |  |  |  |  |  |
| By severity |  |  |  |  |  |  |  |
| Fatal | - |  |  |  |  |  |  |
| Serious | - |  |  |  |  |  |  |
| Not serious | - |  |  |  |  |  |  |
| None | - |  |  |  |  |  |  |
|  |  |  |  |  |  |  |  |
| By site |  |  |  |  |  |  |  |
| Neurological |  |  |  |  |  |  |  |
| Initial stroke |  |  |  |  |  |  |  |
| Complication |  |  |  |  |  |  |  |
| Extension |  |  |  |  |  |  |  |
| Cardiac |  |  |  |  |  |  |  |
| Atrial fibrillation |  |  |  |  |  |  |  |
| Failure |  |  |  |  |  |  |  |
| Hypertension |  |  |  |  |  |  |  |
| Hypotension |  |  |  |  |  |  |  |
| Sudden death |  | - | - | - |  |  |  |
| Gastrointestinal |  |  |  |  |  |  |  |
| Bleed |  |  |  |  |  |  |  |
| Infarction |  |  |  |  |  |  |  |
| Respiratory |  |  |  |  |  |  |  |
| Pneumonia |  |  |  |  |  |  |  |
| PE |  |  |  |  |  |  |  |
| Other |  |  |  |  |  |  |  |
| Unattended death |  | - | - | - |  |  |  |
| DVT |  |  |  |  |  |  |  |
| Malignancy |  |  |  |  |  |  |  |
| Septicaemia |  |  |  |  |  |  |  |
| UTI |  |  |  |  |  |  |  |
| Renal failure |  |  |  |  |  |  |  |
| Other |  |  |  |  |  |  |  |
| … |  |  |  |  |  |  |  |
|  |  |  |  |  |  |  |  |

DVT: deep vein thrombosis; PE: pulmonary embolism; UTI: urinary tract infection

C.7. Protocol violations

Table 7. Protocol violations, by treatment group. Data are number (%).

|  | All | Intensive | Guideline |
| --- | --- | --- | --- |
| As listed in section B.2.8 |  |  |  |
| … |  |  |  |
| Total number of patients |  |  |  |

AF: atrial fibrillation; BP: blood pressure; GCS: Glasgow Coma Scale; TIA: transient ischaemic attack

C.8. Trial flow diagram

Figure 1. CONSORT flow diagram of patient randomisation, outcome, and losses to follow-up. Screening for eligibility was not collected routinely. Data are number (%).

| Randomised |  |  |  |
| --- | --- | --- | --- |
|  | 🡿 |  | 🡾 |
| Treatment allocation | Intensive |  | Guideline |
| Number allocated (safety population) |  |  |  |
| In-patient in hospital |  |  |  |
| Baseline data completed |  |  |  |
| Adherence to allocation | 🡻 |  | 🡻 |
| Any randomised treatment (efficacy population) |  |  |  |
| First dose of randomised treatment |  |  |  |
| > 21 days of randomised treatment |  |  |  |
| All 28/30 days of randomised treatment |  |  |  |
| Day 7 follow-up (during treatment) | 🡻 |  | 🡻 |
| Death by day 7 |  |  |  |
| Day 7 assessment completed |  |  |  |
| <4 days |  |  |  |
| 7-10 days (per protocol) |  |  |  |
| >10 days |  |  |  |
| Day 7 assessment missing |  |  |  |
| Patient refused |  |  |  |
| Logistical problem |  |  |  |
| Other reason |  |  |  |
| Day 35 follow-up (post wash-out) | 🡻 |  | 🡻 |
| Death by day 35 |  |  |  |
| Day 35 assessment completed |  |  |  |
| <30 days |  |  |  |
| 30-40 days (per protocol) |  |  |  |
| >40 days |  |  |  |
| Day 35 assessment missing |  |  |  |
| Patient refused |  |  |  |
| Logistical problem |  |  |  |
| Other reason |  |  |  |
| Hospital discharge or death | 🡻 |  | 🡻 |
| Death in hospital |  |  |  |
| Hospital event form completed |  |  |  |
| Before day 90 follow-up |  |  |  |
| On day 90 follow-up |  |  |  |
| Hospital event form missing |  |  |  |
| Patient refused |  |  |  |
| Logistical problem |  |  |  |
| Other reason |  |  |  |
| Day 90 follow-up (end of follow-up) | 🡻 |  | 🡻 |
| Death by day 90 |  |  |  |
| No vital status available |  |  |  |
| Day 90 assessment completed |  |  |  |
| <83 days |  |  |  |
| 83-97 days (per protocol) |  |  |  |
| >97 days |  |  |  |
| Day 90 mRS assessment missing |  |  |  |
| Patient lost to follow-up |  |  |  |
| Patient refused |  |  |  |
| Logistical problem |  |  |  |
| Other |  |  |  |
|  |  |  |  |

C.9. Distribution of stroke/TIA by its severity

Figure 2. Distribution of stroke/TIA by its severity (fatal / mRS 4-5 / mRS 2-3 / mRS 0- 1 / TIA / no event) at day 90, by treatment group. Comparison by ordinal logistic regression adjusted for baseline factors.

See table 3/primary outcome for source data.

C.10. Outcome by sub-groups

Figure 3. Effect of Intensive vs Guideline antiplatelet therapy on stroke recurrence and its severity at day 90 in subgroups defined prospectively at baseline. Data are odds ratio (95% confidence intervals) and interaction test. Comparison by ordinal logistic regression.

| Variable | N | Intensive | Guideline | OR  (95% CI) | Interaction p |
| --- | --- | --- | --- | --- | --- |
| Geographical region |  |  |  |  |  |
| UK |  |  |  |  | - |
| Other |  |  |  |  | - |
| Age |  |  |  |  |  |
| <75 |  |  |  |  | - |
| >75 |  |  |  |  | - |
| Sex |  |  |  |  |  |
| Female |  |  |  |  | - |
| Male |  |  |  |  | - |
| Index event |  |  |  |  |  |
| Ischaemic stroke |  |  |  |  |  |
| TIA |  |  |  |  | - |
| Clinical syndrome |  |  |  |  |  |
| LACS |  |  |  |  |  |
| POCS |  |  |  |  | - |
| PACS |  |  |  |  | - |
| TACS |  |  |  |  | - |
| NIHSS in stroke |  |  |  |  |  |
| <3 |  |  |  |  | - |
| >3 |  |  |  |  | - |
| ABCD2 in TIA |  |  |  |  |  |
| <5 |  |  |  |  | - |
| >5 |  |  |  |  | - |
| Crescendo TIA |  |  |  |  |  |
| No |  |  |  |  |  |
| Yes |  |  |  |  |  |
| No. of antiplatelets at baseline |  |  |  |  |  |
| 0 |  |  |  |  | - |
| 1 |  |  |  |  | - |
| 2 |  |  |  |  | - |
| Type of randomised comparator |  |  |  |  |  |
| Aspirin/dipyridamole |  |  |  |  | - |
| Clopidogrel alone |  |  |  |  | - |
| Either |  |  |  |  | - |
| Systolic BP |  |  |  |  |  |
| <140 |  |  |  |  | - |
| 141-160 |  |  |  |  | - |
| >160 |  |  |  |  | - |
| Time to randomisation |  |  |  |  |  |
| <12 |  |  |  |  | - |
| 12.1-24 |  |  |  |  | - |
| >24 |  |  |  |  | - |
| Low dose heparin |  |  |  |  |  |
| Yes |  |  |  |  | - |
| No |  |  |  |  | - |
| Thrombolysis |  |  |  |  |  |
| Yes |  |  |  |  | - |
| No |  |  |  |  | - |
| Gastroprotection |  |  |  |  |  |
| Yes |  |  |  |  | - |
| No |  |  |  |  | - |
| Ipsilateral carotid stenosis ¶ |  |  |  |  |  |
| <50 % |  |  |  |  | - |
| >50 % |  |  |  |  | - |
| Old lesion on baseline scan |  |  |  |  |  |
| No |  |  |  |  | - |
| Yes |  |  |  |  | - |
| All patients |  |  |  |  | - |

¶ Usually determined after randomisation

NOTE: This table will be presented as a Forest plot.

C.11. Distribution of modified Rankin Scale

Web Figure 1. Distribution of modified Rankin Scale in all patients at day 90, by treatment group. Comparison by ordinal logistic regression adjusted for baseline factors.

See table 4/secondary outcome for source data.

C.12. Distribution of bleeding by its severity

Web Figure 2. Distribution of bleeding by its severity (fatal / severe / moderate / mild / none) in all patients at day 90, by treatment group. Comparison by ordinal logistic regression adjusted for baseline factors.

See table 5 for source data.

C.13. Distribution of adverse events by their severity

Web Figure 3. Distribution of adverse events by their severity (fatal / serious / not serious / none) in all patients at day 90, by treatment group. Comparison by ordinal logistic regression adjusted for baseline factors.

See table 6 for source data.

C.14. Survival curve

Web Figure 4. Cumulative hazard of death during the 90 days of follow-up after randomisation. Cox proportional regression with adjustment for baseline covariates, hazard ratio= (95% CI - ), p= .

C.15. Meta-analysis of relevant trials

Web Figure 5. Meta-analysis of trials comparing more intensive versus less intensive antiplatelet therapy in patients with acute stroke or TIA, and which included more than 100 patients: effect on recurrent stroke.

This will update ([42](#_ENREF_42), [43](#_ENREF_43))

APPENDIX D. SECONDARY PUBLICATIONS

D.1. PUBLISHED

1. None

D.2. SUBMITTED

1. None

D.3. IN PREPARATION

1. Protocol
   - This follows the structure of ([44](#_ENREF_44))
2. Statistical Analysis Plan (this publication)
   - This follows the structure of ([30](#_ENREF_30))

D.4. PLANNED PUBLICATIONS

D.4.1 Baseline

1. Baseline characteristics
   - This will contain data on all baseline variables and will follow the structure of ([45](#_ENREF_45))
   - See appendix E

D.4.2 Treatment-related

1. Safety and efficacy of intensive versus guideline antiplatelet therapy in patients with acute ischaemic stroke
   - This will follow the structure of the main publication
2. Safety and efficacy of intensive versus guideline antiplatelet therapy in patients with acute transient ischaemic attack
   - This will follow the structure of the main publication
3. Comparison of intensive versus guideline antiplatelets on platelet P-selectin
   - This substudy is using the methodology of ([46](#_ENREF_46))
4. Relationship between presence of headache and stroke recurrence and outcome in patients randomised to dipyridamole
   - This subgroup analysis tests the hypothesis in ([47](#_ENREF_47)) and follows the approach in that publication and a planned secondary analysis listed in the ENOS trial SAP ([30](#_ENREF_30))
5. Comparison of comparators
   - Aspirin/dipyridamole vs clopidogrel alone

D.4.3 ‘Epidemiological’ (not treatment-related)

1. Recurrent events by time to recruitment
   - Type and severity of events

D.4.3 Systematic reviews/meta-analyses

1. Intensive versus guideline antiplatelets
   - This will follow the approach of ([42](#_ENREF_42), [43](#_ENREF_43))

APPENDIX E. BASELINE PAPER

E.1. Enrolment by countries

Table 1. Recruitment: number of sites and patients by region [numbers and %] and country.

| Regions | Countries | No. of sites | No. of patients (%) | % |
| --- | --- | --- | --- | --- |
| Australasia |  |  |  |  |
|  | New Zealand |  |  |  |
| British Isles |  |  |  |  |
|  | UK |  |  |  |
| Continental Europe |  |  |  |  |
|  | Denmark |  |  |  |
|  | Georgia |  |  |  |
| Other regions … | Other countries … |  |  |  |

E.2. Baseline characteristics

Table 2. Baseline characteristics of patients enrolled into the TARDIS trial, by treatment group. Data are number (%), median [interquartile range] or mean (standard deviation).

|  | N | IS | TIA | All |
| --- | --- | --- | --- | --- |
| Number of patients |  |  |  |  |
| Age (years) † |  |  |  |  |
| Sex, male (%) † |  |  |  |  |
| Geographical region ‡ |  |  |  |  |
| Europe, rest of |  |  |  |  |
| UK |  |  |  |  |
| World, rest of |  |  |  |  |
| Source of referral |  |  |  |  |
| Emergency Department |  |  |  |  |
| Outpatient clinic |  |  |  |  |
| Ambulance |  |  |  |  |
| General practitioner |  |  |  |  |
| Inpatient ward |  |  |  |  |
| Other |  |  |  |  |
| Pre-stroke mRS [/6] |  |  |  |  |
| 0 (%) |  |  |  |  |
| 1-2 |  |  |  |  |
| Medical history (%) |  |  |  |  |
| Hypertension, treated ‡ |  |  |  |  |
| Hyperlipidaemia, treated |  |  |  |  |
| Diabetes mellitus |  |  |  |  |
| If diabetes, what drugs |  |  |  |  |
| Antiplatelet agent |  |  |  |  |
| Aspirin |  |  |  |  |
| Aspirin & dipyridamole |  |  |  |  |
| Other |  |  |  |  |
| Atrial fibrillation, current/previous |  |  |  |  |
| Stroke |  |  |  |  |
| Ischaemic heart disease |  |  |  |  |
| Peripheral arterial disease |  |  |  |  |
| Smoking, current (%) |  |  |  |  |
| Qualifying event (%) † |  |  |  |  |
| Ischaemic stroke |  |  |  |  |
| Transient Ischaemic Attack |  |  |  |  |
| Crescendo |  |  |  |  |
| Dual antiplatelets |  |  |  |  |
| Non-stroke |  |  |  |  |
| Side of lesion, right (%) |  |  |  |  |
| Weakness (%) |  |  |  |  |
| Sensory loss (%) |  |  |  |  |
| Dysphasia (%) |  |  |  |  |
| Isolated |  |  |  |  |
| Neglect (%) |  |  |  |  |
| Hemianopia (%) |  |  |  |  |
| Isolated |  |  |  |  |
| National Institute of Health Stroke scale (/42) † |  |  |  |  |
| ABCD2 score [/7] |  |  |  |  |
| Glasgow Coma Scale [/15] |  |  |  |  |
| OCSP classification (%) |  |  |  |  |
| Total anterior † |  |  |  |  |
| Partial anterior |  |  |  |  |
| Lacunar |  |  |  |  |
| Posterior |  |  |  |  |
| TOAST classification (%) Ω∫ |  |  |  |  |
| Cardioembolic |  |  |  |  |
| Large vessel |  |  |  |  |
| Small vessel |  |  |  |  |
| Mixed |  |  |  |  |
| Other |  |  |  |  |
| Haemodynamics |  |  |  |  |
| Systolic blood pressure (mmHg) † |  |  |  |  |
| Diastolic blood pressure (mmHg) |  |  |  |  |
| Heart rate, mean (bpm) |  |  |  |  |
| Brain imaging (%) |  |  |  |  |
| Normal/no lesion |  |  |  |  |
| Ischaemic stroke |  |  |  |  |
| Non stroke |  |  |  |  |
| No brain scan |  |  |  |  |
| Time onset to randomisation [hours] † |  |  |  |  |
| Index event |  |  |  |  |
| Ischaemic stroke |  |  |  |  |
| TIA |  |  |  |  |
| Time (%) |  |  |  |  |
| <12 |  |  |  |  |
| 13-24 |  |  |  |  |
| 25-48 |  |  |  |  |
| >48 ∆ |  |  |  |  |
| Thrombolysis † |  |  |  |  |
| Time to thrombolysis [hours] |  |  |  |  |

† Minimisation variable; ‡ Stratification variable; Ω Ischaemic patients only; ∆ Protocol violation

bpm: beats per minute; NIHSS: National Institutes of Health Stroke Scale; mRS: modified Rankin Scale; OCSP: Oxford Community Stroke Project TOAST: Trial of ORG 10172 in Acute Stroke Treatment

E.3. Trial issues

Table 3. Issues with the trial.

| Issue | Explanation | Comment(s) |
| --- | --- | --- |
| … | … | … |

See example in supplement of ([45](#_ENREF_45))

REFERENCES

1. Weir CJ, Lees KR. Comparison of stratification and adaptive methods for treatment allocation in an acute stroke clinical trial. StatMed 2003;22:705-26.

2. Bamford J, Sandercock P, Dennis M, Burn J, Warlow C. Classification and natural history of clinically identifiable subtypes of cerebral infarction. Lancet 1991;337(8756):1521-6.

3. Rashid P, Weaver C, Leonardi-Bee JA, Fletcher S, Bath FJ, Bath PMW. The effects of transdermal glyceryl trinitrate, a nitric oxide donor on blood pressure, cerebral and cardiac haemodynamics and plasma nitric oxide levels in acute stroke. J Stroke Cerebrovasc Dis 2003;13:143-51.

4. Bath PMW, Geeganage CM, Gray LJ, Collier T, Pocock S. Use of ordinal outcomes in vascular prevention trials: comparison with binary outcomes in published stroke trials. Stroke 2008;39(10):2817-23.

5. Bath PM, Geeganage C, Gray LJ. Ordinal reanalysis of the SHEP trial. Stroke 2008;39(e145).

6. The Optimising Analysis of Stroke Trials (OAST) Collaboration. Should stroke trials adjust functional outcome for baseline prognostic factors? Stroke 2009;40:888-94.

7. Sprigg N, Gray LJ, England T, et al. A randomised controlled trial of triple antiplatelet therapy (aspirin, clopidogrel and dipyridamole) in the secondary prevention of stroke: safety, tolerability and feasibility. PLoS One 2008;3(8):e2852.

8. Geeganage CM, Tracy M, Bath MW, Bath PM. Blood pressure reduction and cardiovascular prevention: meta-regression using ordered categorical (ordinal) event data. J Hypertens 2010;28(10):1995-9.

9. Sare GM, Gray LJ, Bath PMW. Association between hormone replacement therapy and subsequent arterial and venous vascular events: a meta analysis. European Heart Journal 2008.

10. Bath P, Hogg C, Tracy M, Pocock S. Calculation of numbers-needed-to-treat in parallel group trials assessing ordinal outcomes: case examples from acute stroke and stroke prevention. Int J Stroke 2011;6(6):472-9.

11. Rankin J. Cerebral vascular accidents in patients over the age of 60. 2. Prognosis. Scottish Medical Journal 1957;2:200-15.

12. Bath P, Lindenstrom E, Boysen G, et al. Tinzaparin in acute ischaemic stroke (TAIST): a randomised aspirin-controlled trial. Lancet 2001;358:702-10.

13. Lees KR, Bath PMW, Schellinger PD, et al. Contemporary outcome measures in acute stroke research: choice of primary outcome measure Stroke 2012;43(4):1163-70.

14. Committee for Proprietary Medicinal Products. Points to consider on clinical investigation of medicinal poducts for the treatment of acute stroke. London: The European Agency for the Evaluation of Medicinal Products; 2001 20 September 2001. Report No.: CPMP/EWP/560/98 Contract No.: Document Number|.

15. The National Institute of Neurological Disorders and Stroke rt-PA Stroke Study Group. Tissue plasminogen activator for acute stroke. New England Journal of Medicine 1995;333:1581-7.

16. Furlan A, Higashida R, Wechsler L, et al. Intra-arterial prourokinase for acute ischemic stroke. The PROACT II study: a randomized trial. Journal of the American Medical Association 1999;282:2003-11.

17. Hacke W, Albers G, Al-Rwi Y, et al. The Desmoteplase in Acute Ischemic Stroke Trial (DIAS). A phase II MRI based 9-hour window acute stroke thrombolysis trial with intravenous desmoteplase. Stroke 2005;36:66-73.

18. Hacke W, Kaste M, Bluhmki E, et al. Thrombolysis with alteplase 3 to 4.5 hours after acute ischemic stroke. New England Journal of Medicine 2008;359(13):1317-29.

19. Mayer SA, Brun NC, Begtrup K, et al. Recombinant activated factor VII for acute intracerebral hemorrhage. New England Journal of Medicine 2005;352(8):777-85.

20. Sandercock P, Wardlaw JM, Lindley RI, et al. The benefits and harms of intravenous thrombolysis with recombinant tissue plasminogen activator within 6 h of acute ischaemic stroke (the third international stroke trial [IST-3]): a randomised controlled trial. Lancet 2012;379(9834):2352-63.

21. Sandset EC, Bath PM, Boysen G, et al. The angiotensin-receptor blocker candesartan for treatment of acute stroke (SCAST): a randomised, placebo-controlled, double-blind trial. Lancet 2011;377(9767):741-50.

22. Anderson CS, Heeley E, Huang Y, et al. Rapid blood-pressure lowering in patients with acute intracerebral hemorrhage. N Engl J Med 2013;368(25):2355-65.

23. International Stroke Trial Collaborative Group. The International Stroke Trial (IST); a randomised trial of aspirin, subcutaneous heparin, both, or neither among 19435 patients with acute ischaemic stroke. Lancet 1997;349:1569-81.

24. Lees KR, Zivin JA, Ashwood T, et al. NXY-059 for acute ischemic stroke. New England Journal of Medicine 2006;354(6):588-99.

25. Shuaib A, Lees KR, Lyden P, et al. NXY-059 for the treatment of acute ischemic stroke. New England Journal of Medicine 2007;357:562-71.

26. Dengler R, Diener HC, Schwartz A, et al. Early treatment with aspirin plus extended-release dipyridamole for transient ischaemic attack or ischaemic stroke within 24 h of symptom onset (EARLY trial): a randomised, open-label, blinded-endpoint trial. Lancet Neurol 2010;9(2):159-66.

27. Kennedy J, Hill MD, Ryckborst K, et al. Fast assessment of stroke and transient ischaemic attack to prevent early recurrence (FASTER): a randomised controlled pilot trial. Lancet Neurology 2007;6:961-9.

28. Weaver CS, Leonardi-Bee J, Bath-Hextall FJ, Bath PMW. Sample size calculations in acute stroke trials: A systematic review of their reporting, characteristics, and relationship with outcome. Stroke; a journal of cerebral circulation 2004;35(5).

29. The Optimising Analysis of Stroke Trials (OAST) Collaboration. Calculation of sample size for stroke trials assessing functional outcome: comparison of binary and ordinal approaches. International Journal of Stroke 2008;3:78-84.

30. Bath P, Houlton A, Woodhouse L, Sprigg N, Wardlaw J, Pocock S. Statistical analysis plan for the ‘Efficacy of Nitric Oxide in Stroke’ (ENOS) trial. International Journal of Stroke 2014;9(3):372-4.

31. Whynes DK, Sprigg N, Selby J, Berge E, Bath PM. Testing for differential item functioning within the EQ-5D. Med Decis Making 2013;33(2):252-60.

32. The Optimising Analysis of Stroke Trials (OAST) Collaboration. Can we improve the statistical analysis of stroke trials? Statistical re-analysis of functional outcomes in stroke trials. Stroke 2007;38:1911-5.

33. Bath PM, Lees KR, Schellinger PD, et al. Statistical analysis of the primary outcome in acute stroke trials Stroke 2012;43(4):1171-8.

34. Tilley BC, Marler J, Geller NL, et al. Use of a global test for multiple outcomes in stroke trials with application to the National Institute of Neurological Disorders and Stroke t-PA stroke trial. Stroke 1996;27:2136-42.

35. Intravenous Magnesium Efficacy in Stroke (IMAGES) Study Investigators. Magnesium for acute stroke (Intravenous Magnesium Efficacy in Stroke trial): randomised controlled trial. Lancet 2004;363:439.

36. Graeb DA, Robertson WD, Lapointe JS, Nugent RA, Harrison PB. COMPUTED TOMOGRAPHIC DIAGNOSIS OF INTRAVENTRICULAR HEMORRHAGE - ETIOLOGY AND PROGNOSIS. Radiology 1982;143(1).

37. Morgan TC DJ, Spengler D, Lees KR, Aldrich C, Misha NK, Lane K, Quinn TJ, Deiner-Wet M, Weir CJ, Higgins P, Refferty M, Kinsley K, Ziai W, Awad I, Walters MR, Hanley D. The Modified Graeb Score: An enhanced Tool for Intraventricular Hemorrhage Measurement and Prediction of Functional Outcome. Stroke 2013;44:635-41.

38. Schulman S, Kearon C, on behalf of the subcommittee on control of anticoagulation of the scientific and standardization committee of the international society on thrombosis and haemostasis. Definition of major bleeding in clinical investigations of antihemostatic medicinal products in non-surgical patients. Journal of Thrombosis and Haemostasis 2005;3:592-694.

39. Anonymous. Myocardial infarction redefined--a consensus document of The Joint European Society of Cardiology/American College of Cardiology Committee for the redefinition of myocardial infarction. European Heart Journal 2000;21(18):1502-13.

40. Cheung K, Oemar M, Oppe M, Rabin R. EQ-5D User Guide: Basic information on how to use EQ-5D 2ed. <http://www.euroqol.org:> EuroQol Group, 2009.

41. Bath PMW, Woodhouse L, Scutt P, et al. Management of high blood pressure in acute stroke: Efficacy of Nitric Oxide in Stroke (ENOS), a partial-factorial randomised controlled trial. Lancet 2014;In Press.

42. Geeganage CM, Diener HC, Algra A, et al. Dual or mono antiplatelet therapy for patients with acute ischemic stroke or transient ischemic attack: systematic review and meta-analysis of randomized controlled trials. Stroke 2012;43(4):1058-66.

43. Wong KSL, Wang Y, Leng X, et al. Early Dual versus Mono Antiplatelet Therapy for Acute Non-Cardioembolic Ischemic Stroke or Transient Ischemic Attack: An Updated Systematic Review and Meta-Analysis. Circulation 2013.

44. The ENOS Trial Investigators. Glyceryl trinitrate vs. control, and continuing vs. stopping temporarily prior antihypertensive therapy, in acute stroke: rationale and design of the Efficacy of Nitric Oxide in Stroke (ENOS) trial (ISRCTN99414122). International Journal of Stroke 2006;1:245-9.

45. ENOS Investigators. Baseline Characteristics of the 4011 patients recruited into the "efficacy of Nitric Oxide in Stroke (ENOS) trial. International Journal of Stroke 2014;9(6):711-20.

46. Fox SC, May JA, Shah AB, Neubert U, Heptinstall S. Measurement of platelet O-selectin for remote testiing of platelet function during treatment with clopidogrel and/or aspirin. Platelets 2009;20(4):250-9.

47. Davidai G, Cotton D, Gorelick P, et al. Dipyridamole-induced headache and lower recurrence risk in secondary prevention of ischaemic stroke: a post hoc analysis. European Journal of Neurology 2014;Early View (Online).
